# A SCOPING REVIEW OF THE ARTIFICIAL INTELLIGENCE–BASED CONVERSATIONAL AGENTS ON MENTAL HEALTH CARE FOR OLDER ADULTS

**DOI:** 10.1093/geroni/igae098.0831

**Published:** 2024-12-31

**Authors:** Xiayu Chen, Wan Wen

**Affiliations:** University of Illinois Urbana-Champaign, Champaign, Illinois, United States; University of Illinois Urbana-Champaign, Champaign, Illinois, United States

## Abstract

Conversational Agents (CAs) are increasingly applied in the mental health sector, offering scalable and diverse applications from psychoeducation, symptom assessment, to self-management of mental wellness. The recent advancements in Artificial Intelligence (AI), such as the Large Language Model, have further transformed CAs into more sophisticated systems that allow personalized and intelligent interactions. However, most mental health CAs were not originally designed with the unique needs and preferences of older adults in mind. This oversight highlights the necessity for tailored features that better accommodate older adults’ user experience. Current scholarly research and interventions that focus on AI-based CAs for older adults are scarce. To address this gap, this scoping review aims to offer a comprehensive overview of existing mental health AI-based CA for older adults. We conducted an extensive systematic literature search across thirteen databases, including PubMed, Scopus, IEEE Explore, ACM Digital Library, Web of Science and others. After a thorough selection process, nine peer-reviewed articles were identified for review. Our preliminary findings underscore the usability and effectiveness of AI-based CAs in enhancing the mental well-being of older adults, showing an overall positive user satisfaction and a capability for therapeutic intervention. Nevertheless, challenges such as the need for increased technological literacy and accessible designs specifically tailored for older adults persist. These findings provide critical insights for researchers and practitioners into how AI-based CAs, as promising and accessible applications and interventions, can be optimized to meet the unique mental health needs of older adults.

